# Treatment of inoperable hepatocellular carcinoma with pegylated liposomal doxorubicin (PLD): results of a phase II study

**DOI:** 10.1038/sj.bjc.6602394

**Published:** 2005-02-08

**Authors:** J W Valle, A Dangoor, J Beech, D J Sherlock, S M Lee, J H Scarffe, R Swindell, M Ranson

**Affiliations:** 1 Christie Hospital NHS Trust, Manchester, Wilmslow Road, Manchester M20 4BX, UK; 2 The Pennine Acute Hospitals NHS Trust, North Manchester General Hospital, Delaunays Road, Crumpsall, Manchester M8 5RB, UK; 3 UCLH, The Middlesex Hospital, Mortimer Street, London W1T 3AA, UK; 4 East and North Herts NHS Trust, Lister Hospital, Coreys Mill Lane, Stevenage, Herts SG1 4AB, UK

**Keywords:** pegylated liposomal doxorubicin, hepatocellular carcinoma, phase II

## Abstract

Monthly intravenous pegylated liposomal doxorubicin (PLD) 50 mg m^−2^, although well tolerated, showed almost no activity in this phase II study of 16 patients with advanced hepatocellular carcinoma with a response rate of 0%, stable disease 19%, median time to progression of 2.4 months, 1-year survival of 25% and median survival of 6.5 months.

Surgery, currently the only treatment offering long-term cure, is possible in a minority of patients with hepatocellular carcinoma (HCC) (Llovet *et al*, 2003). In selected patients with unresectable, liver-only, disease hepatic embolisation or chemoembolisation confers a survival advantage compared with conservative management (Llovet *et al*, 2002; Lo *et al*, 2002). However, in the recent meta-analysis, this effect may be limited to chemoembolisation but not embolisation (Llovet and Bruix, 2003). Chemotherapy for inoperable and extrahepatic disease is relatively ineffective; the current ‘standard’, doxorubicin, has a response rate of 16% (range 0–35%) in a review of eight trials including 475 evaluable patients (Engstrom *et al*, 1993). It is associated with a small survival advantage over supportive care alone in a randomised study of 106 chemotherapy-naïve patients at the expense of significant toxicity (mucositis, alopecia, myelosuppression and cardiotoxicity) (Lai *et al*, 1988).

Liposomes, capable of encapsulating anticancer agents without chemical modulation, accumulate preferentially in the reticuloendothelial system (Weinstein, 1984). This uptake may be evaded by ‘stealth’ liposomes (formed by altering the liposome lipid composition), which preferentially exit the circulation via leaky capillaries and are predicted to accumulate in tumours exhibiting extensive neo-vascularisation (such as HCC) leading to higher concentrations and enhanced antitumour activity (Wu *et al*, 1993). Pegylated liposomal doxorubicin (PLD), an intravenous preparation of doxorubicin hydrochloride encapsulated in long-acting pegylated ‘stealth’ liposomes, can avoid rapid hepatic uptake resulting in a prolonged circulation time. Compared to conventional doxorubicin, intratumoural doxorubicin levels in tumour-bearing mice were substantially higher when using PLD. Moreover, enhanced cytotoxicity of an HCC cell line has been demonstrated using ‘stealth’ liposomes compared to conventional doxorubicin (Shimizu *et al*, 1996).

Dose-limiting toxicities of PLD include reversible neutropaenia, stomatitis and plantar-palmar erythrodysaesthesia. Alopecia and emesis are infrequent (Uziely *et al*, 1995). A phase I study in 33 patients with advanced HCC determined neutropaenia as the dose-limiting toxicity at 60 mg m^−2^ and recommended 50 mg m^−2^ every 28 days as the phase II dosing regimen (Schering Plough – data on file). Endomyocardial biopsies have shown PLD to have a superior cardiac safety profile compared to nonliposomal doxorubicin (Berry *et al*, 1998).

## MATERIALS AND METHODS

### Patient selection

An open-label, phase II study was performed. Chemotherapy-naïve patients (including chemotherapy as part of chemoembolisation procedures) with histologically proven HCC, aged ⩾18 years, with measurable disease by CT/MRI scan (WHO criteria), WHO performance score (PS) of ⩽2, life expectancy ⩾3 months, left ventricular ejection fraction (LVEF) ⩾50% (by MUGA or echocardiography), haemoglobin >9 g dl^−1^, white blood cells ⩾3.0 × 10^9^ l^−1^, neutrophils ⩾1.5 × 10^9^ l^−1^ and platelets ⩾100 000 mm^−3^, serum creatinine <1.5 × upper limit of normal (ULN), bilirubin <1.5 × ULN and AST/ALT <2 × ULN (unless related to primary disease when value <5 × ULN) were eligible. Patients with New York Heart Association⩾Class II cardiac disease, patients who were pregnant, lactating or were unwilling to use contraception during the study were excluded. The appropriate Local Research Ethics Committees at each institution approved the study and all patients gave written informed consent prior to study entry.

### Treatment

Patients received PLD (Caelyx®/Doxil®, Schering-Plough headquarters, Kenilworth, NJ, USA) 50 mg m^−2^ in 250–500 ml of 5% dextrose i.v. over 1 h on day 1 of each 28-day cycle. Retreatment occurred upon recovery of platelets to ⩾75 000 mm^−3^, ANC to ⩾1.5 × 10^9^ l^−1^ and skin toxicity or stomatitis to grade ⩽1. A 25% dose reduction was permitted for delayed recovery from toxicity or grade ⩾2 toxicity. Patients were withdrawn from the study for a deferral of a treatment cycle (for toxicity) by more than 2 weeks.

### Evaluation

Tumour assessments (CT/MRI scan) and cardiac assessments (ECG/LVEF) were made at baseline and after cycles 3, 6 and 9. Patients received PLD until disease progression, unacceptable toxicity, patient choice or physician's discretion. Toxicity (NCI-Common Toxicity Criteria, version 2.0), vital signs and serum biochemistry (including serum AFP) were assessed at baseline and at every cycle with weekly blood counts during the study period. All patients were followed up for survival data.

## RESULTS

A total of 16 patients (see Table 1 for patient characteristics) received a total of 47 cycles (median 3, range 1–9) at two institutions (Christie Hospital, Manchester, UK (*n*=13) and University College Hospital, London, UK (*n*=3)) between November 1998 and April 2002.

### Toxicity

Treatment was well tolerated with most toxicities reported at grade 1. Grade 3–4 toxicity included leucopaenia, neutropaenia, nausea/vomiting and lethargy in 12, 12, 6 and 6% of patients, respectively (and 4, 6, 2 and 2% of cycles, respectively). Dose delays were required on three occasions and a total of three dose reductions were made. One patient with persistent grade 4 neutropaenia was withdrawn from treatment. No cardiac toxicity was observed. Hepatic toxicity was mostly grade 1; grade 2 hyperbilirubinaemia was seen in 27% of patients (9% of cycles) and elevated serum alkaline phosphatase in 9% of patients (3% of cycles). No grade 2 transaminitis or grade 3–4 hepatotoxicity was documented (Table 2 ).

### Response and survival

No responses were seen in the first 16 patients and the study was closed to accrual as the minimal threshold of activity had not been reached according to the Simon one sample, two-stage design (Simon, 1989). Three patients (19%) had stable disease after three cycles, with disease progression in the remaining patients (including the three patients with FLHCC). One of the three patients with stable disease had ongoing stable disease after six and nine cycles of treatment. The median time to disease progression in all patients was 2.4 months with 1-year and 2-year survival rates of 25 and 12.5%, respectively. The median survival of all patients was 6.5 months. As expected, patients with FLHCC had a longer median survival (20.5 months) than patients with standard HCC histology (4.3 months).

Second-line chemotherapy was administered to six patients (38%) (gemcitabine alone (*n*=3) or with cisplatin (*n*=3)). Two patients receiving cisplatin–gemcitabine achieved disease stabilisation having previously progressed, the remainder had disease progression. One patient with FLHCC achieved a sustained partial response with continuous-infusion 5-FU and interferon having failed PLD followed by cisplatin–gemcitabine and remains alive at 54 months.

Two patients (13%) with disease confined to the liver were deemed suitable for chemoembolisation at first assessment. The choice was made to proceed with PLD in the first instance (prior chemoembolisation would have deemed them ineligible). Both progressed after three cycles of PLD and then underwent chemoembolisation (achieving survivals of 16 and 25 months, respectively). Five patients (31%) received palliative hormone therapy.

## DISCUSSION

Doxorubicin, with modest activity, is a toxic agent and patients with advanced HCC are frequently unwell due to their underlying tumour and hepatic dysfunction. There was an expectation that its efficacy could be maintained (or even enhanced) with the better tolerated PLD formulation (Uziely *et al*, 1995).

A number of studies contemporary to ours (see Table 3 ) have shown, at best, response rates of 10–17% (Ruff *et al*, 2001; Schmidinger *et al*, 2001; Hong and Tseng, 2003). Two studies, as in our case, revealed no responses: in the first, no objective responses were seen in 16 patients receiving 30–40 mg m^−2^ three-weekly. With a median survival of 20 weeks, the authors concluded that PLD was ineffective (Halm *et al*, 2000). Miller *et al* (2002) reached the same conclusion with a 0% response rate in a mixed study of 10 HCC (and eight cholangiocarcinoma) patients. Based on a median survival of 1-year, Schmidinger *et al* (2001) concluded that PLD warranted further studies. However, Ruff *et al* (2001) who demonstrated a 17% response rate achieved a median survival of only 5.3 months. The difficulty in relying on response rate as a marker of activity was further illustrated by Hong and Tseng (2003): despite 10% of patients responding to treatment and a further 33% achieving stable disease, the median survival was only 3 months.

A favourable toxicity profile has been consistent across all the HCC studies (Halm *et al*, 2000; Ruff *et al*, 2001; Schmidinger *et al*, 2001; Miller *et al*, 2002; Hong and Tseng, 2003). We found few grade 3–4 toxicities and notably none involving palmar-plantar erythrodysaesthesia, a dose-limiting toxicity seen in early studies along with stomatitis and neutropaenia (Muggia *et al*, 1997; Ranson *et al*, 1997). Cardiac toxicity was not seen and dose reductions or dose delays due to toxicity were uncommon. Enhanced activity of PLD was postulated by increased leakage from capillaries in vascular tumours once the drug had evaded rapid hepatic uptake. This reduced uptake may, however, also be responsible for the lack of hepatotoxicity and, ultimately, efficacy.

Despite the ability to deliver doxorubicin more safely to patients, PLD has almost no activity in advanced HCC. This reflects the relative insensitivity of HCC to chemotherapy agents and success may come from alternative treatment modalities aimed at better targeting of the underlying pathological processes involved in HCC carcinogenesis.

## Figures and Tables

**Table 1:** Patient demographics

**Characteristics**	**Number (%)**
Patients entered	16
Age (years)	
Median (range)	56 (23–65)
	
Sex	
Male	12 (75%)
Female	4 (25%)
	
Performance status at enrolment (WHO)	
0	3 (19%)
1	13 (81%)
	
Tumour histology	
Hepatocellular carcinoma	13 (81%)
Fibrolamellar type	3 (19%)
	
Tumour stage	
4A	7 (44%)
4B	9 (56%)

**Table 2:** Reported toxicity by patient (*n*=16) and by cycle (*n*=47)

	**By patient**				**By cycle**			
**Toxicity/grade**	**1**	**2**	**3**	**4**	**1**	**2**	**3**	**4**
Haemoglobin	63	13	0	0	47	6	0	0
WBC	13	6	6	6	13	6	2	2
Neutrophils	13	13	6	6	15	4	4	2
Platelets	25	0	0	0	17	2	0	0
Nausea/vomiting	63	6	6	0	45	2	2	0
Hepatic								
Bilirubin	36	27	0	0	26	9	0	0
Alkaline phosphatase	27	9	0	0	32	3	0	0
AST	55	0	0	0	29	0	0	0
								
Diarrhoea	38	6	0	0	19	15	0	0
Lethargy	31	31	6	0	34	9	2	0
Cutaneous	25	0	0	0	28	0	0	0
Anorexia	19	6	0	0	11	2	0	0
Fever/sepsis	13	13	0	0	6	4	0	0
Mucositis	13	13	0	0	4	9	0	0
Alopecia	0	6	0	0	0	6	0	0
Constipation	0	6	0	0	0	2	0	0
Cardiac	0	0	0	0	0	0	0	0

**Table 3:** Contemporary studies of PLD in advanced HCC

**Study**	***N***	**Dose**	**RR (%)**	**s.d. (%)**	**MS (months)**	**Reference**
Halm *et al* (2000)	16	30–40 mg m^−2^ q3wks	0		4.6	Halm *et al* (2000)
Miller *et al* (2002)	10	50 mg m^−2^ q4wks	0	30	5.0	Miller *et al* (2002)
Valle *et al* (current study)	16	50 mg m^−2^ q4wks	0	19	6.5	
Schmidinger *et al* (2001)	17	40 mg m^−2^ q4wks	14	36	12	Schmidinger *et al* (2001)
Ruff *et al* (2001)	42	50 mg m^−2^ q4wks	17		5.3	Ruff *et al* (2001)
Hong *et al* (2003)	40	30–45 mg m^−2^ q3wks	10	33	3	Hong and Tseng (2003)

PLD=pegylated liposomal doxorubicin; HCC=hepatocellular carcinoma.
